# A Hydrophilic 3D-Printed Microfluidic Device for Emulsion Studies: Preliminary Observations on the Role of Naphthenic Acids in Coalescence

**DOI:** 10.1021/acsomega.5c05121

**Published:** 2025-11-05

**Authors:** Lucas Paines Bressan, Reverson Fernandes Quero, Millena Couto dos Santos, Rogério Mesquita de Carvalho, Leandro Wang Hantao

**Affiliations:** † Instituto de Química, Universidade Estadual de Campinas, Campinas, São Paulo 13083-862, Brasil; ‡ Centro de Pesquisas Leopoldo Américo Miguez de Mello (CENPES), Petrobras, Rio de Janeiro, Rio de Janeiro CEP 21.949-900, Brasil; § Instituto Nacional de Ciência E Tecnologia (INCTBio), Campinas, São Paulo 13083-862, Brasil; ∥ Centro de Estudos de Energia E Petróleo (CEPETRO), Campinas, São Paulo CEP 13083-896, Brasil; ⊥ Núcleo Interdisciplinar de Planejamento Energético (NIPE), Campinas, São Paulo CEP 13083-896, Brasil

## Abstract

Produced water (PW) comes from oil production and contains both nonpolar and polar substances that can be dissolved or dispersed in the aqueous phase. Such a complex mixture is typically found as an oil/water emulsion (O/W), which can be stabilized by the presence of interfacial materials, such as naphthenic acids (NAs). Recent literature has focused on isolating and characterizing the acidic interfacial material, but our fundamental understanding of the role of NAs in emulsion stabilization and coalescence remains scarce. This is justified by the challenges associated with droplet formation in microfluidics. For instance, the high viscosity of the O/W affects the dominant forces in the pinch-off, as well as wetting conditions due to surface chemistry compatibility with O/W, thereby limiting the efficiency of droplet generation. In addition, most reported devices rely on external actuation to induce coalescence, with few design options currently available. In this context, enabling technologies are desperately needed to probe the coalescence of complex O/W systems and to allow for the correlation of compositional data at the molecular level with the stability of O/W. In this study, a 3D-printed microfluidic device was created using an LCD-based SLA printer and a custom hydrophilic and transparent resin suitable for coalescence studies with O/W. The device features channels of ∼40 μm for consistent droplet generation and coalescence observation without requiring surface modifications or external actuation to induce coalescence. The device was tested using a model O/W system, demonstrating controlled droplet generation and observation of coalescence phenomena. The effect of NAs on emulsion stability was investigated by analyzing mixtures of NAs from both crude oil and produced water. It was found that NAs inhibited droplet coalescence, which was consistent with their role as surfactants, and reduced interfacial tension. Comprehensive two-dimensional gas chromatography coupled with high-resolution mass spectrometry (GC×GC-HRMS) was used to characterize the molecular composition of NAs in the samples for further interpretation of the coalescence data. The GC×GC-HRMS results revealed differences in carbon number distribution and degree of cyclization of NAs, allowing for correlation with observed variations in emulsion stability. This study showed that 3D-printed devices can be engineered for droplet generation and coalescence investigations using a single, disposable device. This report is particularly important in showcasing the new and upcoming applications of inexpensive (less than 1 US dollar) polymeric 3D-printed devices under harsh environments that would otherwise cause substrate swelling.

## Graphical abstract

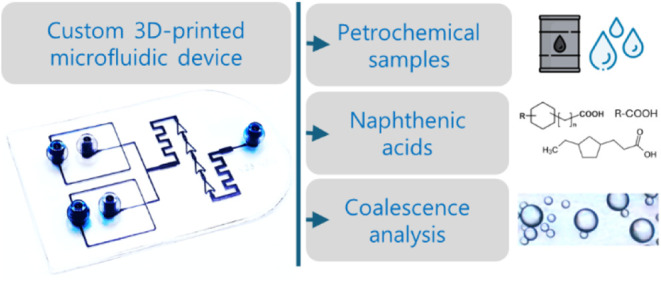

## Introduction

Produced water (PW) is generated during oil production, and large amounts can be either reinjected into the well to recover oil or discharged into the environment.^1^ The PW is considered a complex matrix, containing a range of soluble and insoluble matter, requiring it to be treated before reinjecting into the well or discharging.^2^ One of the major components of PW is oil in the form of an oil/water emulsion (O/W). In the case of offshore processing, the grease content is regulated to prevent problems related to oil discharge and oil sheen formation. Specifically for reinjection, damage formation can occur if the oil concentration is not low enough. In either case, treatment of PW is a key factor in the petroleum industry, and understanding its composition is required to optimize its reuse or disposal.^3^

Naphthenic acids (NAs) are a complex mixture of carboxylic acids naturally present in crude oil and produced water. NAs are considered natural surfactants and play an important role in emulsion stabilization due to their amphiphilic nature.^4–6^ In case of O/W, these compounds can adsorb at the interface when in salt form, reducing interfacial tension and promoting the formation of stable emulsions resistant to coalescence, which can hinder the removal of oil content from PW.^7^ The molecular structure of NAs, especially their molecular weight and degree of cyclicity, influences their solubility and distribution between the aqueous and oil phases, thereby affecting the characteristics of the emulsion.^8,9^

A detailed study of the coalescence of oil droplets in water is fundamental to understanding the mechanisms that govern emulsion stability and, consequently, for developing strategies to control and optimize produced water treatment in the petroleum industry.^10,11^ In this case, microfluidics is a suitable tool for studying emulsions, allowing precise manipulation of small fluid volumes and the creation of controlled environments for emulsion generation and analysis.^12^ Studying coalescence under microfluidic conditions provides a deeper understanding of the underlying phenomena and parameters that affect the samples.^13^ However, traditional microfluidic devices, usually fabricated from glass or silicon, present geometric and material limitations that hinder coalescence investigations in complex samples like crude oil or PW. These limitations include chemical compatibility, difficulty in fabricating complex geometries, and the need for surface treatments to adjust the hydrophilic or hydrophobic properties of the channel’s surface.

To overcome such limitations, three-dimensional (3D) printing emerges as a promising solution, offering different printing techniques,^14,15^ an extensive range of materials and the option to fabricate devices with customized geometries quickly and at low cost. One of the uses of 3D-printed microfluidic devices is the generation of stable emulsions, since they can be customized with the appropriate geometry, using either filament-based techniques^16^ or resin-based techniques.^17–19^ However, these devices are not optimized for the study of coalescence within microfluidic chips, possibly due to limitations such as large channel sizes (>50 μm) and insufficient transparency to visible light. To enable handling and observation of emulsions inside microfluidic devices, it is necessary to develop specific materials that are transparent and possess appropriate hydrophilic properties for efficient droplet generation.

Our group previously reported the use of 3D printing to manufacture disposable microfluidic devices for the sample preparation of petroleum, successfully overcoming the well-known limitations of nonsilicon and nonglass microchips.^20^ Aspects such as solvent swelling and the conservation of sample integrity were effectively addressed using the proposed approach. However, the two processes involved in this microchip, namely droplet generation and coalescence, introduce an entirely different set of parameters that challenge 3D printing, including the chemical composition of the resin and the design of the microfluidic device. For instance, challenges in droplet formation within microfluidic systems include the impact of high O/W viscosity on dominant pinch-off forces, which affects droplet uniformity and generation efficiency.^21^

Most microfluidic chips require surface treatments or coatings to achieve the appropriate hydrophilicity, which complicates fabrication and potentially introduces chemical heterogeneities that affect reproducibility.^22^ Additionally, reported devices typically depend on external actuation methods, such as mechanical, electrical, or acoustic stimuli, to induce droplet coalescence, limiting experimental design versatility.^23,24^

Commercially available chip designs for droplet studies are limited to a few options, and 3D-printed microfluidic chips present limitations, such as large channel dimensions (typically greater than 100 μm) and limited optical transparency, which restricts precise observation of droplet interactions and thus limits coalescence analysis.^25^ Consequently, there is demand for customized microfluidic devices with optimized geometry, inherent hydrophilicity, chemical inertness, and improved optical transparency for coalescence studies.

While microfluidic devices have advanced the study of coalescence phenomena by enabling precise control over emulsion generation and droplet interactions,^26^ the complex chemical composition of petrochemical samples necessitates the integration of complementary analytical techniques for comprehensive analysis.^27,28^ To address these limitations, combining microfluidic coalescence studies with advanced analytical methods, such as two-dimensional gas chromatography coupled with high-resolution mass spectrometry (GC×GC-HRMS), enables the detailed characterization of surface-active compounds, like naphthenic acids, that influence emulsion stability.^29,30^ This holistic approach provides further insight into the relationship between the chemical composition and coalescence behavior of petroleum samples, which is necessary for developing effective strategies to enhance PW treatment and reduce the formation of O/W emulsions.

In this work, we present a novel, customized microfluidic device 3D-printed using a liquid crystal display (LCD)-based stereolithography (SLA) printer with specialized resin, which overcomes these limitations and provides the necessary characteristics for studying coalescence in produced water samples. The device is transparent, hydrophilic, and resistant to organic solvents, allowing for the generation and analysis of O/W under controlled conditions. The resin used eliminates the need for additional modifications to adjust the hydrophilicity of the channels, simplifying fabrication. The developed device was tested using a model system, where a model mixture was used to produce emulsions, allowing for the observation and analysis of coalescence processes using a high-speed camera. Subsequently, studies were conducted with mixtures of NAs from crude oil and produced water, obtaining information on the coalescence behavior of the samples. Samples were also characterized by GC×GC-HRMS, allowing a thorough characterization of the NAs present in the samples. To the best of our knowledge, this work is the first to develop a LCD-based SLA 3D-printed polymeric microfluidic device with inherently hydrophilic surfaces and closed channels designed explicitly for generating O/W droplets and studying their coalescence in the presence of NAs without the use of external actuators.

## Material and Methods

### Reagents and Materials

Technical mixture of naphthenic acids (Sigma-Aldrich – São Paulo, Brazil) was used as received. Ultrapure water (Type 1) was obtained from a Milli-Q IQ7003 system (Merck – São Paulo) with a resistivity of 18.2 MΩ·cm. Isopropanol and ethanol (Neon, São Paulo – Brazil) were reagent grade.

### Oil and Produced Water Samples

Petroleum and PW samples were provided by the Leopoldo Américo Miguez de Mello Research Center (CENPES) of the Brazilian Petroleum Company (Petrobras – Rio de Janeiro, RJ).

### Liquid–Liquid Extraction (LLE)

Methylene chloride (HPLC-grade) and sodium sulfate (Synth – São Paulo, SP, Brazil) were used in the LLE procedure. The method was developed by our research group.^30,31^

First, the pH of the PW samples was adjusted to 2 with a 3.5% (v/v) HCl solution. An aliquot of 15 mL of sample was extracted with 10 mL of dichloromethane. This procedure was repeated 3 times, and the three aliquots were combined. Sodium sulfate was added to the extracts to remove any residual water. The extracts were concentrated in a rotary evaporator (Heidolph – Schwabach, Germany) at 40 °C under reduced pressure of 300 mbar. Finally, the dry extract of the samples was dissolved with 2 mL of isooctane.

### Solid-Phase Extraction (SPE)

Methanol, chloroform, and acetone with HPLC-grade purity were employed in the SPE procedure. Sep-Pak Vac RC SPE cartridges with silica sorbent phase modified with “-NH_2_” groups (500 mg) (Waters Corporation – Milford, MA, USA) were used. The SPE method was developed based on the work of Rowland et al.^32,33^ and adapted as follows.

The method consisted of applying an aliquot of 400 mg of oil, diluted in 3 mL of chloroform, onto an SPE cartridge that had been previously conditioned with 15 mL of chloroform. The sample was left in contact with the sorbent phase for 15 minutes to favor the sorption of the acids. The neutral and nonpolar fraction was eluted with 43 mL of chloroform. For the elution of the polar fraction, 20 mL of acetone was used, followed by 10 mL of a mixture of acetone and methanol 1:1 (v/v), and finally another 10 mL of methanol. The fraction containing the NAs was concentrated in a rotary evaporator at 40 °C and reduced pressure of 200 mbar. The final volume of the extract was adjusted to 2 mL with isooctane.

### Derivatization Reaction Using Silylation Reagent

For the derivatization of the NAs, 40 μL of a mixture of *N*-*tert*-butyldimethylsilyl-*N*-methyltrifluoroacetamide (MTBSTFA) and 1% (v/v) *tert*-butyldimethylchlorosilane (t-BDMCS) were added to a 2 mL vial containing an aliquot of 1.0 mL of the polar compounds extract. The vial was immediately sealed and subjected to stirring (250 rpm) at 70 °C. More information is available in the article published by our research group.^34^ After the derivatization step, the samples were analyzed using the GC×GC method described below.

### Group-Type Analysis of NAs by GC×GC-HRMS

Analyses were performed on a TRACE 1310 gas chromatograph hyphenated to a Q Exactive GC (FT-Orbitrap) mass spectrometer (Thermo Fisher Scientific – Waltham, MA, USA). The GC was equipped with a split/splitless (SSL) injector and a TriPlus RSH autosampler.

The SSL injector operated with a 10:1 split at 300 °C. Helium was used as the carrier gas at a constant flow rate of 1.0 mL/min. A nonpolar × midpolarity column configuration was used. For the first dimension (^1^D), an Rxi 1MS column (30 m × 0.25 mm i.d. × 0.25 μm film thickness) (Restek Corporation – Bellefonte, PA, USA) was used. For the second dimension (^2^D), an Rxi 17Sil MS column (2 m × 0.1 mm i.d. × 0.1 μm film thickness) was used. The oven was held at 100 °C for 1 min, followed by heating to 247 °C at 3 °C/min and to 300 °C at 20 °C/min. The oven was maintained at 300 °C for 2 min.

Modulation was performed using the ZX2 modulator (Zoex Corporation – Houston, TX, USA). A 4 s modulation period with a hot pulse of 250 ms was used in all analyses.

The Q Exactive GC mass spectrometer was used for data acquisition. An acquisition range of 50 Da to 500 Da was used in Full MS mode. A mass resolution of 7,500, measured at 200 Da, was used for spectrum acquisition. The transfer line was maintained at 300 °C, and the electron ionization source was operated at 250 °C. The injection time (IT) and automatic gain control (AGC) parameters were set to automatic mode and 1 × 10^6^, respectively. Additional information can be found in the article published elsewhere.^35^

### Data Processing and NAs Identification Using Scripts

The raw chromatograms were acquired in Xcalibur software (Thermo Fisher Scientific) in “.RAW” format and converted to “.CDF” using the file conversion tool of the same program.

The GC×GC chromatograms were processed in GC Image 2020r3 software (Zoex Corporation), where the total ion chromatograms were processed. The identification of NAs in oil and PW samples was performed using the CLIC (Computer Language for Identifying Chemicals) function of the GC Image software. Detailed scripts are found in the article published by our research group.^34^

### 3D Printing and Device Fabrication

Autodesk Fusion software (student version, 2024) (Autodesk – San Rafael, California) was used to design the 3D-printed microfluidic device, and the drawing was exported as an STL file. Simplify3D software (version 4.1) (Blue Ash, OH, USA) was used to slice and control the printing parameters, resulting in G-code commands for the 3D printer.

An LCD-based SLA 3D printer Phrozen Mini 4K was used, with 35 μm XY resolution and 772 pixels per inch (ppi) (Phrozen, phrozen3d.com). The main parameters used to print the microfluidic device were: normal exposure time: 4 s; base exposure time: 20 s; base layers: 10; wait time between normal layers: 0.5 s; Z-lift distance: 8 mm; Z-lift speed: 2 mm/s; layer height: 0.02 mm; and printing time of approximately 1 h. The resin used for printing the device was specifically developed for this application and has a proprietary composition developed by Polaris Microsystems & Nanotechnology (Campinas, SP – Brazil, polarisnano.com.br). While the formulation is proprietary, it is based on a hydrophilic, photocurable polymer matrix that utilizes poly­(ethylene glycol) methacrylate as one of its oligomers (patent pending).

After printing, the device’s channels were carefully washed by introducing solvents using a syringe pump at a flow rate of 200 μL/min, with the following solvents: 4 mL of isopropanol, 4 mL of ethanol, and 4 mL of a 50:50 (v/v) ethanol:water solution. All solvents were of reagent grade. The devices were washed for 20 minutes with each solvent and then dried with a nitrogen flow for 5 minutes. After drying, the device was subjected to postcuring for 1 minute in an Anycubic Wash and Cure Plus machine (Anycubic – Shenzhen, China) and was then ready for use.

### Microfluidic System

The segmented phase consisted of mineral oil diluted in hexadecane, 20% (w/w) (Sigma-Aldrich – São Paulo, SP, Brazil). Aqueous solutions were provided by CENPES. Synthetic saline water samples were used as the continuous phase, and their compositions are indicated in Table S1 Supporting Information. The pressure used for manipulating the fluids was maintained at 80 mbar for the continuous phase and 5 mbar for the segmented phase.

The liquids were pumped using OB1MK4+ microfluidic flow control modules from Elveflow (Elvesys – Paris, France). The pressure level in the system was controlled by a back-pressure regulator (AF1 Pressure Controller) and monitored with pressure sensors (MSP-EXP) at the chip’s inlets and outlets. The flow was measured with a flow meter (MFS2). The droplets were observed with a Chronos 2.1-HD high-speed camera (Kron Technologies – Burnaby, BC, Canada), connected to a Ti–U Eclipse inverted microscope (Nikon – Japan) with an HDF7010 external LED light source (Hayashi – Japan).

A series of aqueous solutions was prepared to evaluate the coalescence phenomenon in the presence of naphthenic acids by dissolving mixtures of NAs in saline matrices (Table S1). The naphthenic acids were obtained from a Technical Mixture (Sigma-Aldrich) and extracts derived from crude oil and produced water, provided by CENPES. The technical mixture was prepared at a concentration of 3.3 mg/L. The NAs isolated from real-world PW and oil samples had concentrations of 0.9 mg/L and 5.5 mg/L, respectively. These values were determined based on the average total concentration of acids monitored over the past two years. A total of six aqueous samples were prepared and used for the coalescence study using microfluidics. This procedure enabled us to assess the impact of the chemical diversity of NAs on the stability of O/W emulsions.

### Data Acquisition and Image Analysis

High-speed camera analysis was performed using a 10× objective lens, capturing images at 1000 fps with a resolution of 1024 × 1024 pixels. The capture time used for each analysis was 5 s, resulting in a total of 5000 frames. Images of the initial and final chambers for each sample were acquired within a 20-s interval. All data were obtained in raw format (.raw) from the camera and converted to the appropriate format using a custom script. The resulting video was used in.avi format for analysis with the Automated Droplet Measurement software,^36^ obtaining the droplet count and size determination. The obtained data were organized into histograms and analyzed using Origin 2023b Student software (OriginLab – USA). To provide a quantitative measure of emulsion stability, a Droplet Growth Factor (DGF) was calculated. The DGF represents the percentage increase in the mean droplet diameter as the emulsion moves from the initial to the final observation chamber. It was calculated for each experimental condition using the following formula:

1 $$\mathrm{DGF}\;\left(\%\right)=\left(D_{\mathrm{final}}-D_{\mathrm{initial}}\right)/D_{\mathrm{initial}}\times 100$$

where *D*_initial_ and *D*_final_ are the mean equivalent droplet diameters measured in the initial and final visualization chambers, respectively. The calculations were based on the mean diameter of the entire droplet population. A higher DGF value indicates a greater degree of coalescence.

## Results and Discussion

### Development of a Custom 3D-Printed Microfluidic Device

Over the past decade, 3D printing has emerged as an alternative to conventional microfabrication technologies, such as soft lithography, due to its capabilities for customizing microfluidic device designs.^37^ Fused deposition modeling (FDM)-based devices have been less expensive and easier to produce, allowing scientists across various fields to experiment with different applications, such as chemical synthesis and droplet generation. However, FDM-based 3D-printed devices often lack the necessary resolution and transparency and tend to have rougher channel surfaces, which limit their effectiveness in microfluidic applications.^38^

In contrast, light-based 3D printers, such as SLA and digital light processing (DLP) printers, offer higher resolution and smoother surfaces but have traditionally been associated with higher acquisition and operational costs. Additionally, there was a lack of suitable resins tailored for producing the small channel sizes required for microfluidics, further hindering their use.^39^ This situation has changed in recent years with the popularization of LCD-based SLA 3D-printers. These printers are more affordable and capable of producing high-quality microfluidic devices, especially when printing parameters are carefully optimized. The availability of specialized resins compatible with LCD-based printers has also facilitated the fabrication of microfluidic devices with the fine features necessary for advanced applications.^40^ However, limitations exist. Analyzing petroleum samples requires materials that are inert and do not interact with target analytes,^41^ and the range of resins capable of withstanding extreme chemical conditions is limited.^20^ Additionally, the resolution and accuracy of printed objects depend heavily on the quality of the resin, which can be affected by postprocessing to remove uncured resin. Regarding the generation of oil-in-water emulsions, the surface of the 3D-printed microfluidic channel must be hydrophilic, typically requiring external modification through chemical treatments or coatings, which adds complexity to the fabrication process. Finally, high-quality printers and resins can also be cost-prohibitive for some research budgets.

We utilized custom-developed resin, with proprietary composition, designed specifically for microfluidic applications of O/W emulsions using an LCD-based SLA 3D-printer to fabricate the microfluidic device. This resin offers several advantages over commercially available options, including production of hydrophilic channels without postprocessing, enhanced breakage resistance, and resistance to organic solvents such as toluene and xylene. During device development, several commercial resins were tested but failed to meet all required criteria. Data regarding some commercial resins are available in Table S3. Some hydrophilic resins resulted in fragile structures prone to breaking, especially at connection points. Others lacked suitable channel dimensions or hydrophilicity, necessitating surface modifications. Figure 1 presents the final 3D-printed microfluidic device and the microfluidic channels used to generate droplets with channel dimensions of approximately 42 μm.

**Figure 1:**
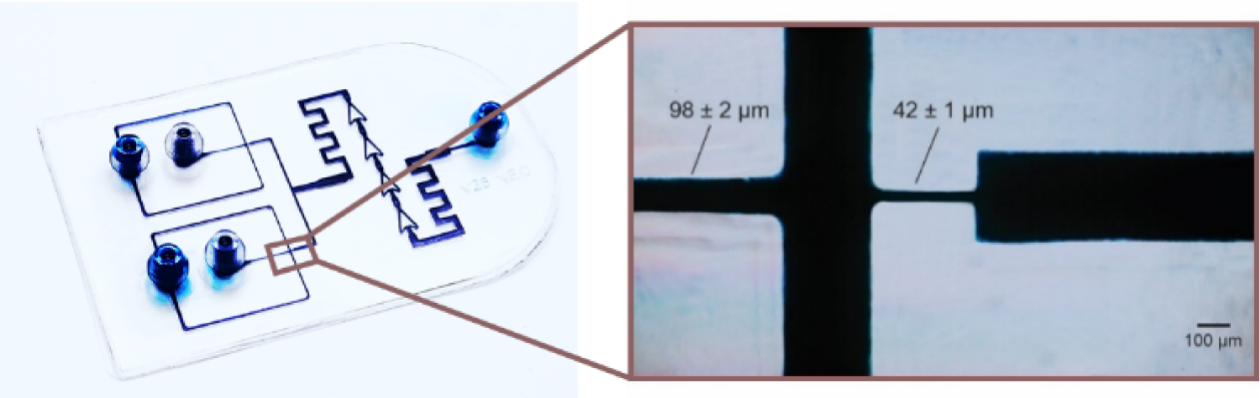
Left: Developed 3D-printed microfluidic device with channels filled with colorant for better visualization of microchannels. Right: Microfluidic channels filled with colorant displaying the region of flow focusing used for droplet generation.

The custom-made resin was tested for solvent sorption to ensure maximum chemical compatibility. Printed pieces (15 mm × 15 mm × 3 mm) were immersed in various solvents and solutions for 24 h. Weight measurements taken before and after immersion were used to assess any changes in the resins’ chemical stability. The studied pieces resulted in weight increase values lower than 0.2% for all tested solvents, except for acetone, where the weight increase was approximately 2.3%. The detailed results are shown in Table S3. The devices were also tested for mechanical resistance, where devices measuring 4 cm × 2.5 cm × 2 mm were subjected to force at the center while supported at the sides. The hydrophilic resin withstood a force of up to 51.5 N before breaking, as shown in Figure S1a.

The 3D-printed devices were also subjected to deflection tests shown in Figure S1b, where deflection angles measured the flexibility of the resins under stress, with the developed resin resulting in a deflection angle of 157.38°, indicating lower flexibility, due to polar monomers resulting in more rigid networks and a matrix less effective at supporting high mechanical loads.

To demonstrate that the 3D-printed device was indeed hydrophilic for generating O/W emulsions, contact angle tests were performed, yielding a 37° contact angle, as shown in Figure S2a.

The letters “PETR” were 3D-printed at a microscale with details smaller than 50 μm, and the result is shown in Figure S2b. Achieving such a resolution depends on the quality of the resin, high-resolution printers, and optimized printing parameters.

Transparency is a key requirement for 3D-printed microfluidic devices, especially in applications involving coalescence studies. For the 3D-printed devices, visible light transmittance (VLT) was measured, and the results are shown in Figure S2c, resulting in an 85.5% VLT, compared to borosilicate glass (1 mm thick) at 92.5% VLT. These results demonstrate that the 3D-printed devices possess optical properties comparable to those of glass, making them suitable for applications that require transparency, such as microscopic observation and reaction monitoring. This control enables customization of surface properties to meet the specific needs of various industries.

### Microfluidic Device Design

In coalescence studies, commercial microfluidic devices are commonly used due to their accessibility and standardized configurations.^1^ However, they often suffer from limitations in geometry and materials, with fixed channel dimensions and layouts that restrict experimental customization, and materials like glass or PDMS that may lack the necessary chemical resistance, mechanical strength, or optical clarity for complex fluids or harsh environments. In contrast, 3D-printed custom-made microfluidic devices overcome these limitations by enabling rapid fabrication of personalized geometries tailored to specific research needs. However, most of the 3D-printed devices proposed in the literature present large channel dimensions (usually more than 100 μm), lack optimized optical transparency, and do not offer solutions for studying coalescence.

The 3D-printed microfluidic device developed in this paper possesses all the characteristics listed above for both O/W formation and coalescence observation. A comparison with some reports in the literature is provided in Table S4. Figure 2 displays each region of the device, with several specific geometries highlighted. A droplet generation area using flow-focusing (Figure 2A), an initial chamber (Figure 2B) for droplet observation, a combination of fluid channels that guide the continuous and segmented phase through the device (Figure 2C andF), two regions designed to reduce turbulency of the flow and allow for droplet collisions and promote coalescence (Figure 2D and E), and a final chamber (Figure 2G) for coalescence observation.

**Figure 2:**
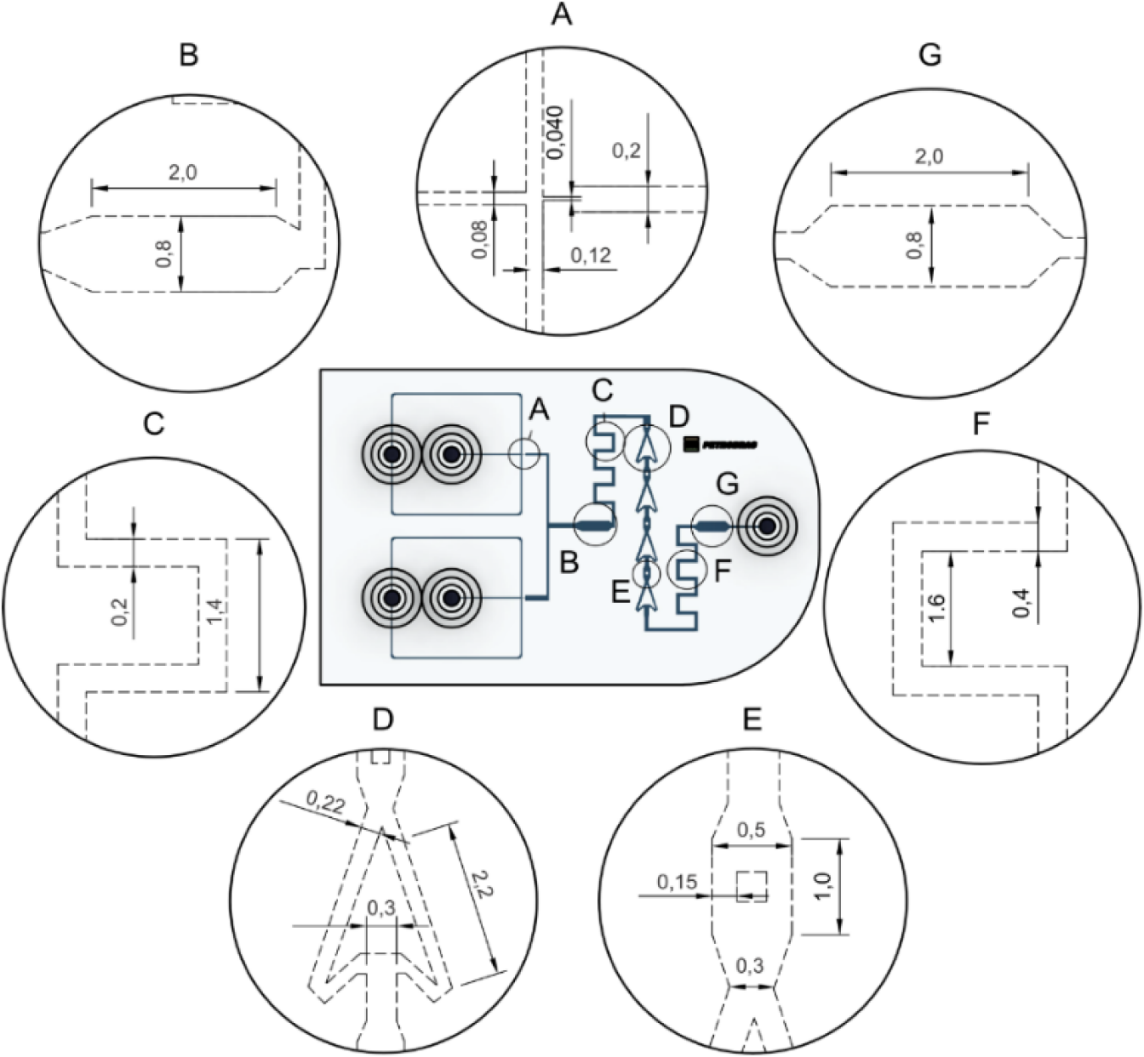
Technical drawing of the developed 3D-printed microfluidic device. Each letter indicates a specific region. A) Droplet generation area with flow focusing. B) Initial visualization chamber. C and F) Serpentine channels for droplet movement and organization. D and E) Fluidic region to direct droplet collision. G) Final visualization chamber. All measurements are in millimeters.

Two primary geometries for droplet formation are used in microfluidics: T-junction and flow focusing. T-junctions are simple but can suffer from the slug effect, leading to nonuniform droplet sizes due to droplet dragging before separation, which is often observed in commercial devices. The flow-focusing geometry relies on a dispersed phase centered by two continuous phases, resulting in controlled and uniform droplet formation.^42^ Hence, the proposed device incorporates a dual-region flow-focusing configuration, enhancing droplet production capacity for comprehensive analyses, as detailed in Figure 3. Figure 3a displays a photograph of the 3D-printed device with the two flow-focusing regions highlighted. Figure 3b shows a schematic representation of the channel design. Figure 3c presents a microscopic image of the oil droplet generation inside the device.

**Figure 3:**
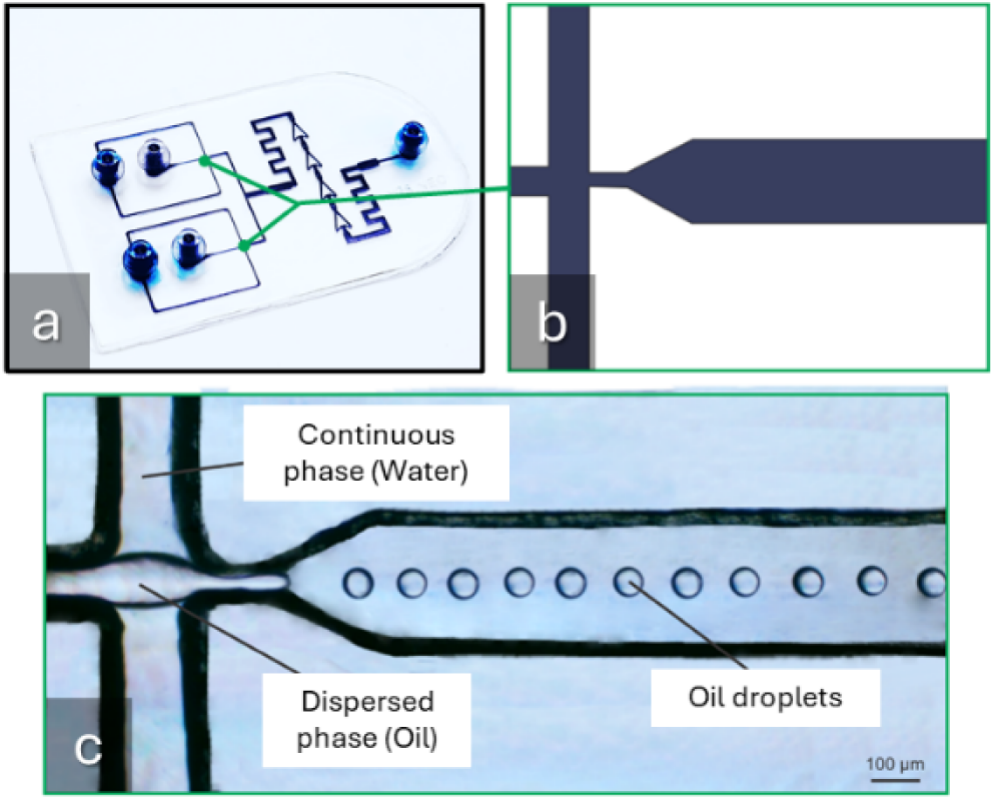
a) Photograph of the 3D-printed microfluidic device with channels filled with colorant for better visualization. b) Schematic drawing of the oil-in-water droplet generator. c) Optical microscopy image of the 3D-printed microfluidic device showing the generation of oil-in-water droplets at the flow-focusing region.

The device incorporates several key features to facilitate the study of droplet coalescence. Two visualization chambers are strategically placed to capture moments of the coalescence process through high-speed video recording. Figure 4a shows the 3D-printed device and both visualization chambers: initial (Figure 4b), where the oil droplets have a uniform equivalent diameter, and final (Figure 4c), where the coalescence phenomena are observable due to the larger droplet sizes present. These features enable the precise manipulation and observation of droplet dynamics, providing insight into the mechanisms that govern emulsion stability. Additionally, it includes a separation region where droplets are initially isolated and accelerated, allowing controlled collisions between them and a coalescence-inducing region, where the droplets are decelerated and allowed to coalesce. More details are shown in Figure S3.

**Figure 4:**
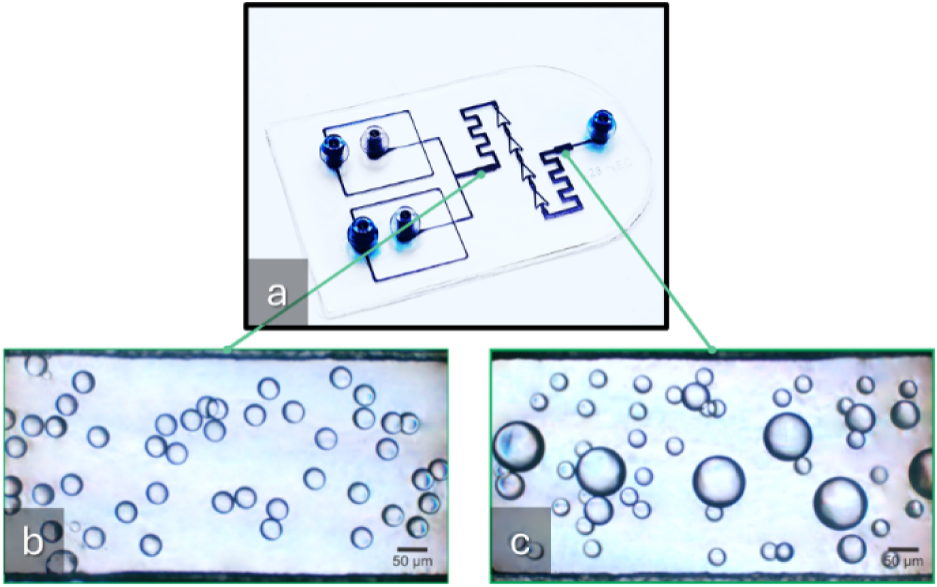
a) Photograph of the 3D-printed microfluidic device with channels filled with colorant for better visualization, indicating two regions for droplet observation. b) Optical microscopy image of the initial coalescence chamber displaying generated oil-in-water emulsion with droplet sizes of similar equivalent diameter. c) Optical microscopy image of the final coalescence chamber displaying generated oil-in-water emulsion (O/W) with droplet sizes of varying equivalent diameter, indicating coalescence of the generated droplets.

To further evaluate the device fabrication toward droplet formation, ultrapure water was used as the continuous phase, and hexadecane solution was used as the segmented phase, representing the oil fraction, resulting in the generation of droplets with controlled sizes and a size dispersion of approximately 1% of the mean diameter. Figure S4 presents histograms of droplet equivalent diameter distributions for four distinct regions of the device (generation zone A and B, initial coalescence chamber C, and final coalescence chamber D). Panels A and B display narrow diameter distributions with a mean value of 27 μm, indicating homogeneous particle sizes. Panel C shows a slightly broader distribution with decreasing frequencies as the diameter increases. In Panel D, a broader peak shifted toward larger diameters is observed, evidencing droplet coalescence where smaller particles merge to form larger ones, thus validating the device’s design effectiveness. Figure S5 presents a frame-by-frame analysis of two oil droplets merging for further demonstration of the applicability of the proposed device. A corresponding video is provided in the Supporting Information.

### Microfluidic Analysis of Coalescence of Real Samples

After evaluating the device’s ability to generate O/W with controlled droplet sizes, it was possible to assess the effect of NAs on emulsion stability. Previous works have already investigated the role of such compounds in O/W emulsions, using commercially available microfluidic devices^1^ or custom-designed microfluidic devices,^43,44^ both are using glass as the substrate. However, the indication for such devices is that both a cleaning step and a surface modification step are necessary to utilize the devices, which are not needed in our case with the fabricated 3D-printed device. The experimental setup used a solution of hexadecane as the segmented oil phase and solutions of the technical mixture of NAs in ultrapure water as the continuous aqueous phase. The results are shown in Figure 5.

**Figure 5:**
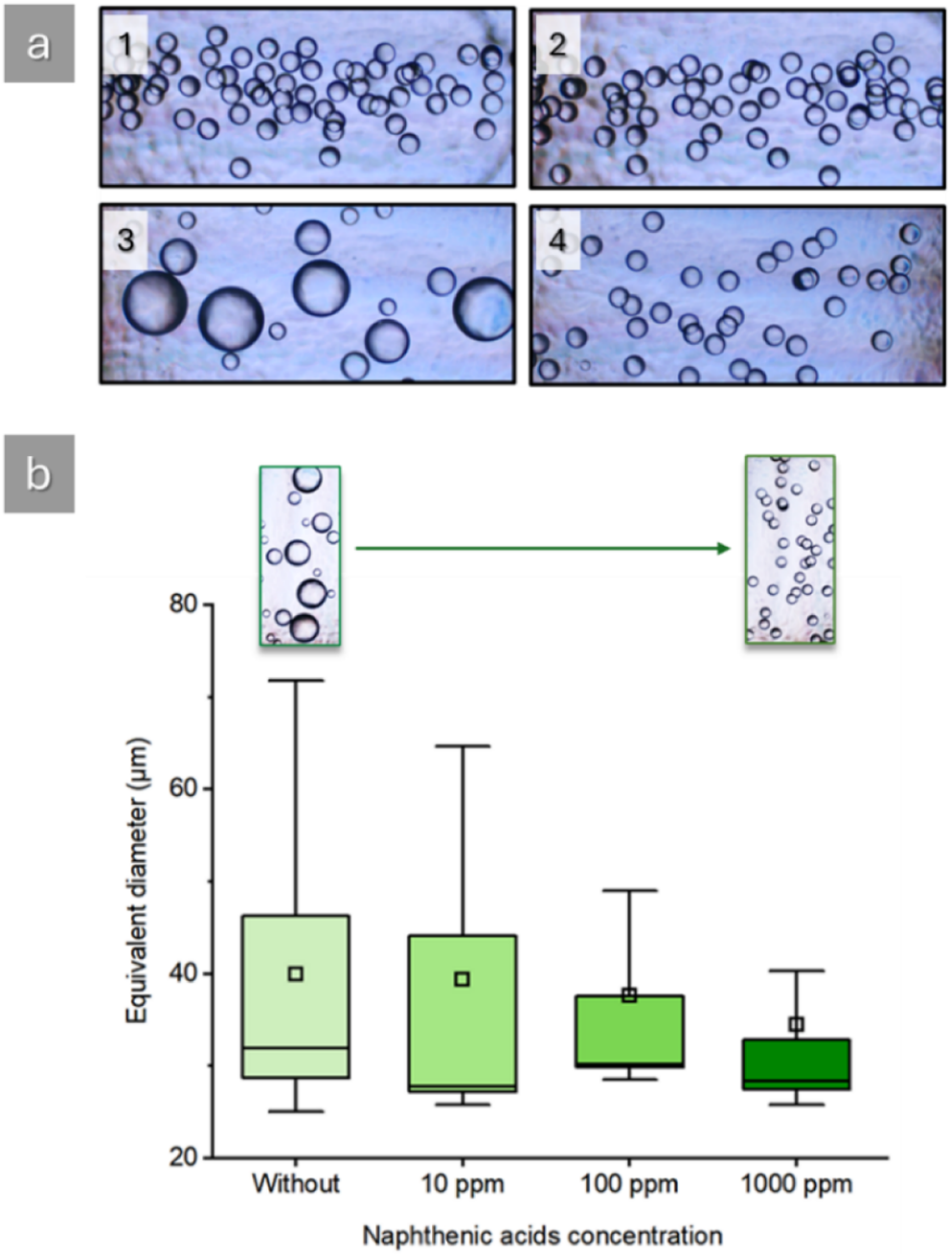
a) Optical microscopic image showing the impact of naphthenic acids (NAs) on the coalescence of oil droplets in water. The initial (1 and 2) and final (3 and 4) observation chambers display the dispersion of oil droplets in the absence of NAs (1 and 3) and the presence of NAs (2 and 4). The comparison between the final coalescence chambers highlights the effect of NAs on the coalescence of droplets, where in the presence of NAs the effect of coalescence is not observed. b) Boxplot analysis of the equivalent diameter of oil droplets under different naphthenic acid concentration conditions: “Without” indicates the absence of naphthenic acids, and the subsequent labels represent increasing concentrations of naphthenic acid at 10 ppm, 100 ppm, and 1000 ppm. The interquartile range (whiskers), median (line), and mean (square) are marked. Microscopic images demonstrate the reduction of the coalescence phenomenon.

Analysis of microscopic images suggests that naphthenic acids affect the coalescence of oil droplets in water, as seen in Figure 5a. In both initial and final observation chambers where NAs are present, oil droplets maintain their size and dispersion. Data was obtained from both initial and final coalescence chambers with a difference of less than 20 s between measurements. This time is necessary to move the microscope lens and allow the system to equilibrate. In contrast, in the absence of NAs, the droplets coalesce, as indicated by the increase in droplet size in the final observation chamber. Additional information is presented in Figure 5b, where box-plot graphs provide further details regarding the size distribution of the samples. The analysis reveals that the mean (represented by the square) and median equivalent diameters (represented by the line) of the droplets decrease with increasing NAs concentration, suggesting enhanced emulsion stabilization. Moreover, DGF analysis resulted in a 33.8% increase for the solution without NAs and a 13.3% increase for the solution with 1000 ppm of NAs from the technical mixture, indicating an increased stabilization of the oil droplets in solution when NAs are present.

Previous works stated that at lower concentrations, NAs act as surfactants, reducing interfacial tension and stabilizing droplets against coalescence.^7,45^ However, as the concentration increases, additional NAs do not contribute to further stabilization once the interface becomes saturated.^45^ The decrease in droplet size and variability from 0 to 1000 ppm (w/v) indicates that NAs at higher concentrations continue to effectively stabilize the droplets, preventing them from coalescing back into larger sizes. This suggests the existence of an optimal concentration range for NAs to achieve maximum stabilization efficiency. Beyond this range, the effect of NAs on stabilization reaches a plateau or potentially introduces other complexities, such as overstabilization, which can be detrimental for specific applications.^4^ Therefore, although naphthenic acids are important for determining droplet stability, an optimal concentration appears to exist beyond which no significant stabilization gains are observed, as seen in the comparison between the effects at 100 ppm and 1000 ppm.

After evaluating the device with a model system, we investigated its potential in analyzing real samples containing naphthenic acids extracted from produced water and crude oil. Each sample extract was diluted in two saline waters with different compositions (Table S1), resulting in four aqueous samples being used as the continuous aqueous phase. At the same time, hexadecane solution served as the dispersed oil phase. Two additional solutions were prepared with the technical mixture. This procedure was developed to assess the effect of pH value and the chemical diversity of NAs on the stability of O/W emulsions. The results obtained for the samples are presented in Figure 6 as histograms of the equivalent diameter values for the oil droplets and their median size at the specified intervals.

**Figure 6:**
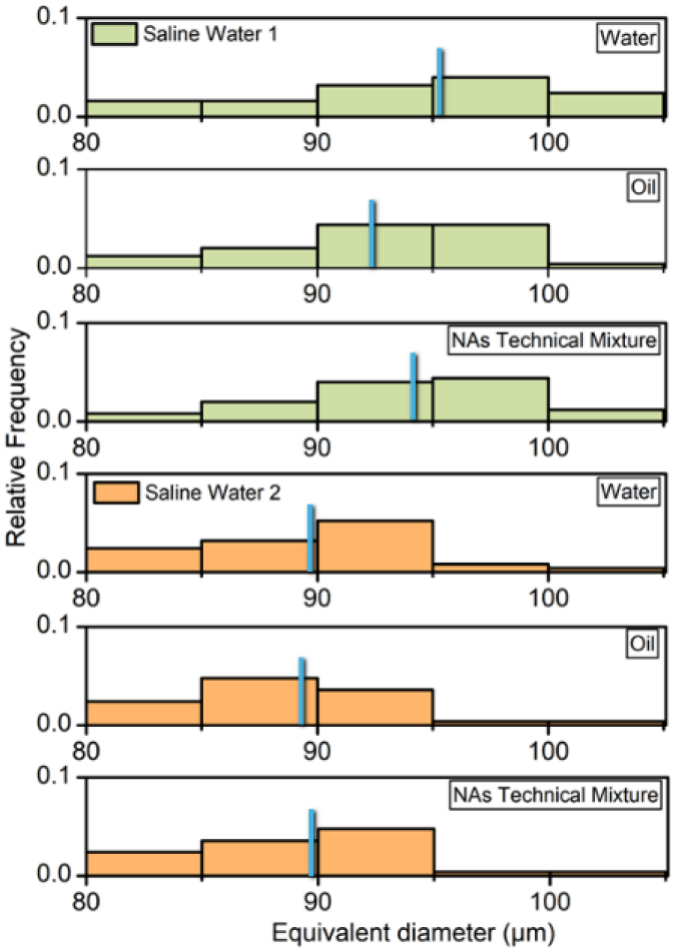
Histograms obtained for the different samples analyzed in the microfluidic device. The distributions start at 80 μm to highlight the coalescence phenomenon at higher equivalent diameter values. The blue bar represents the median value in the interval presented.

The results led to the following observations. Samples prepared in saline water 1 (SW1, pH 7.4) showed more frequent equivalent diameters in the region between 90 and 100 μm. In contrast, the equivalent diameters for samples prepared in saline water 2 (SW2, pH 8.3) are in the region between 85 and 90 μm. DGF measurements for SW1 were 18.5%, 18.1% and 19.0% for water, oil, and technical mixture samples, respectively. For SW2, DGF measurements obtained were 12.3%, 11.3% and 11.8% for water, oil, and technical mixture samples, respectively. Additionally, distinctions among samples prepared in the same saline water were observed as follows: oil samples showed higher frequency regions in the histogram for lower equivalent diameter values in both SW1 and SW2. Conversely, produced water samples presented results with higher equivalent diameter values, notably in SW1, where the highest equivalent diameter values were found. The technical mixture of naphthenic acids showed results between those observed for the samples derived from produced water and oil. These behaviors can be attributed to interfacial tension effects and surfactant dynamics between the NAs and the emulsion.^44^ Initially, emulsions are produced by generating dispersed droplets in a continuous phase—in this system, oil droplets in water. One way to measure the stability of an emulsion system is through interfacial tension (IT), which measures the force acting at the interface between two immiscible liquids. High IT values indicate emulsion instability because they increase the force that separates the liquid phases, facilitating the coalescence of oil droplets. The decrease in IT values can be achieved by adding surfactants, which are molecules responsible for generating stable droplets and preventing the coalescence of newly formed droplets.^46^ In this case, NAs act as surfactants in oil-in-water systems by decreasing the IT of oil droplets.

Despite providing information on coalescence behavior, the proposed technique using the 3D-printed microfluidic device can benefit from more data regarding the chemical composition of the samples studied. This approach enabled us to analyze the molecular composition of the NAs in the original PW and crude oil samples using GC×GC-HRMS. This analytical technique provided detailed information on the carbon number distribution and degree of cyclization (double bond equivalent, DBE) of the NAs present. The GC ×GC-HRMS results showed that the PW sample predominantly contained NAs with carbon numbers distributed around 10 and DBE values close to 2. In contrast, the crude oil sample exhibited a NAs distribution centered at a carbon number of 15 and a DBE of 3, as seen in Figure 7.

**Figure 7:**
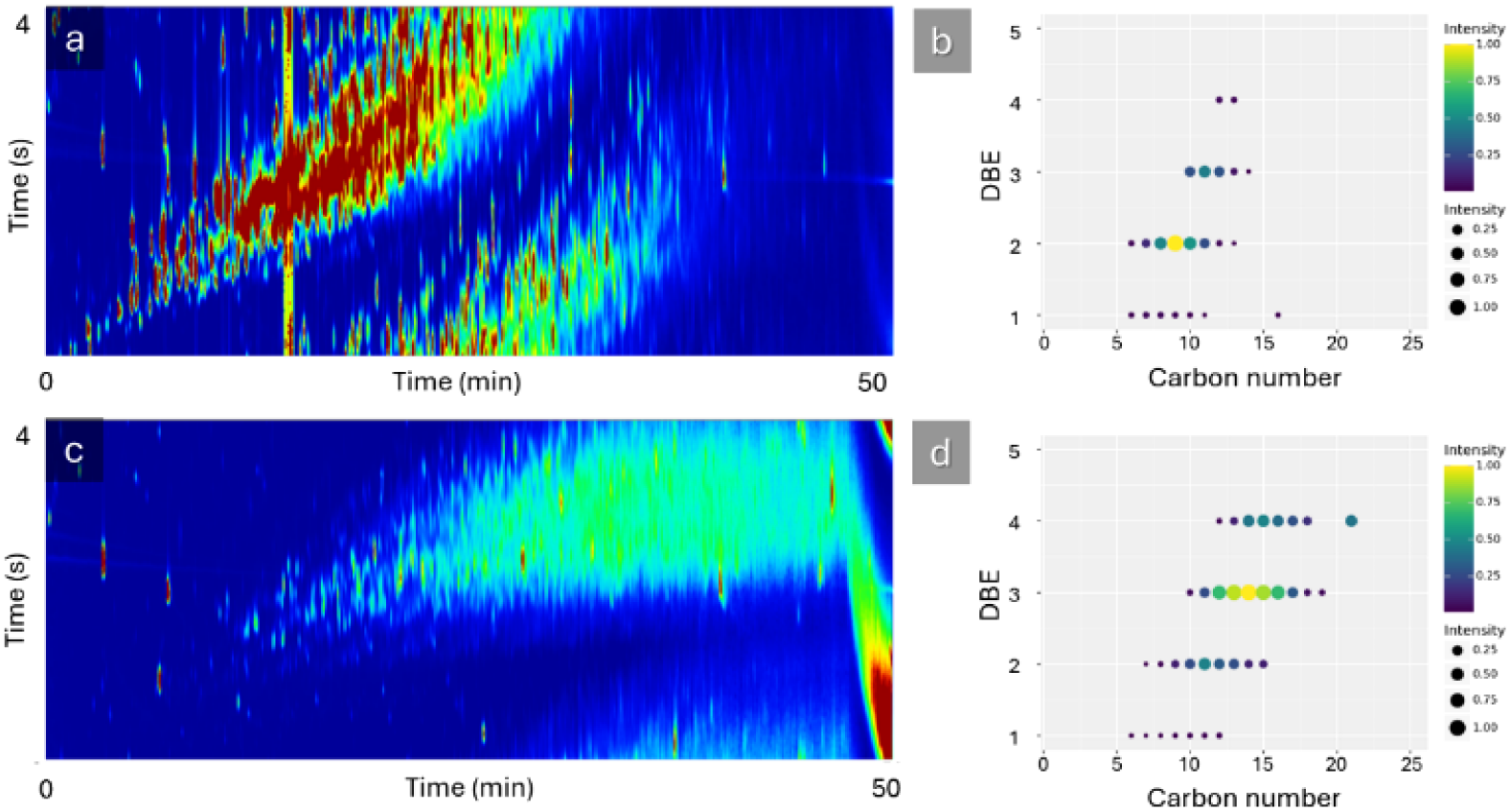
a) GC×GC-HRMS chromatogram for the analyzed produced water sample. b) Comparison between double bond equivalence (DBE) and carbon number for the naphthenic acids found in the water sample. c) GC×GC-HRMS chromatogram for the analyzed crude oil sample. d) Comparison between DBE and carbon number for the naphthenic acids found in the crude oil sample. For the DBE vs carbon number, the intensity is represented by both the color and the size of the circles.

The stability of O/W emulsions is influenced by the aqueous phase chemistry and the molecular structure of NAs at the phase interface. The observed differences in coalescence, particularly the enhanced stability in SW2 (pH 8.3) compared to SW1 (pH 7.4), can be explained through an analysis of NA ionization, electrostatic forces, and the specific structural contributions of the NAs identified by GC×GC-HRMS. The primary mechanism controlling NA activity is the pH of the aqueous phase, which dictates the degree of ionization of the carboxylic acid head groups. NAs are weak acids with a reported p*K*_a_ in the range of 4.9–6 (data for selected NAs is presented in Table S5). At the pH values used in this study (7.4 and 8.3), a significant portion of the NAs are deprotonated into their anionic naphthenate form, which is the primary surface-active species responsible for stabilizing the O/W emulsion. The adsorption of anionic naphthenates onto the surface of the oil droplets imparts a net negative charge, creating an electrical double layer that generates a repulsive energy barrier, preventing droplet coalescence.^45,48^ The superior stability in emulsions observed in SW2 (pH 8.3) is a direct consequence of this principle. At this higher pH, which is further from the p*K*_a_ of the NAs, the acid–base equilibrium shifts more strongly toward the ionized naphthenate form. This leads to a higher surface charge density on the droplets, resulting in stronger electrostatic repulsion that hinders coalescence, as evidenced by the droplet diameters observed in the final chamber for all samples in SW2 (Figure 6) and the overall lower values of DFG obtained compared to SW1.

Conversely, at the lower pH of 7.4 in SW1, the degree of NA ionization is reduced. This results in a lower surface charge density and weaker electrostatic repulsion, making the droplets more susceptible to coalescence when they collide within the microfluidic device. Furthermore, at this lower pH, the less soluble, neutral NAs are more abundant. These species, particularly those with longer carbon chains and anionic naphthenates, can aggregate or precipitate at the interface in the presence of divalent cations (Ca^2+^, Mg^2+^) found in the saline waters, forming solid-like films. This can lead to a different stabilization mechanism, one that resembles a Pickering emulsion, where solid particles stabilize the interface.^49,50^ However, such films can be poorly organized compared to the electrostatic barrier formed at higher pH, thus providing a less effective barrier against the dynamic stresses within the microfluidic channels and leading to the greater coalescence observed in SW1.

Moreover, the influence of the molecular structure of the NAs is another key factor, as revealed by the GC×GC-HRMS analysis (Figure 7). The crude oil extract, which consistently produced the most stable emulsions, is rich in NAs with higher carbon numbers (centered at 15) and a greater degree of cyclicity (DBE centered at 3). Longer aliphatic chains (carbon number >14) are known to increase the viscoelasticity of the interfacial film, providing a more robust mechanical barrier that resists the drainage and rupture required for coalescence.^47^ The higher degree of cyclicity (related to DBE) also plays a significant role. It is believed that NAs containing multiple rings can pack more efficiently at the interface, forming highly ordered, rigid films or even liquid-crystalline phases that physically hinder droplet merging.^51^ In contrast, the NAs from the produced water sample, with their lower carbon number (carbon number ∼ 10) and lower cyclicity (DBE ∼ 2), form a less cohesive and more fluid interfacial layer, offering less protection against coalescence. Therefore, the observed stability trends are a synergistic result of both chemical environment and molecular structure. The highest stability, observed with the crude oil extract in SW2, is attributed to the combination of electrostatic repulsion (resulting from the high pH) and a superior physical barrier (provided by NAs with high carbon numbers and DBE). The lowest stability, observed with the PW extract in SW1, is a result of weaker electrostatic forces and a less effective molecular structure. This integrated analysis reinforces the necessity of combining microfluidic observations with detailed chemical characterization, such as GC×GC-HRMS to fully elucidate the complex mechanisms governing emulsion stability in petrochemical systems.^52^

## Conclusions

A 3D-printed microfluidic device was successfully developed using a resin appropriate for coalescence studies in O/W. The device offers the chemical compatibility and optical properties required for analyzing petrochemical samples without surface modifications, particularly in the generation of O/W and the coalescence of oil droplets.

This inexpensive device costs less than a US dollar and was devised to be expandable. Conversely, more expensive glass chips (over USD 500) require extensive cleaning between experiments to minimize changes in surface chemistry, which inevitably affect droplet generation and, thus, negatively impact longer-term coalescence studies. Furthermore, a significant improvement over existing literature was the design of the microchip, which bypassed the need for external actuation to induce droplet coalescence.

Using this device, the effect of naphthenic acids on emulsion stability was investigated. Naphthenic acids act as surfactants, reducing interfacial tension and stabilizing emulsions against coalescence. Coalescence studies were conducted with real-world samples and combined with comprehensive two-dimensional gas chromatography coupled with high-resolution mass spectrometry, providing molecular compositions of naphthenic acids and their influence on emulsion behavior. Both the concentration and structural features of naphthenic acids affect emulsion stability, indicating that integrating detailed molecular analysis with coalescence studies is necessary for understanding emulsion phenomena in petrochemical systems.

This investigation focused on a specific crude oil and produced water system, and its purpose was not to cover all aspects of emulsion stability. Instead, the aim was to elucidate the applicability of the integrated approach, demonstrating how detailed molecular analysis can provide further data along the coalescence phenomena observed in the microfluidic device. These findings provide the necessary initial information and a proof-of-concept, suggesting that this combined methodology can be used to develop a deeper understanding of emulsion behavior in complex petrochemical samples, which is relevant for developing strategies to mitigate industry challenges such as injectivity loss.

## Supplementary Material





## Notes

Additional information on mechanical tests performed on the hydrophilic resin; characterization tests on the 3D-printing resin; schematic diagram of microfluidic device regions; particle size distribution histograms; time series images of droplet coalescence; composition of saline waters for the coalescence study; weight increase measurements after immersion; comparison to commercially available resins; review of the literature regarding the generation and coalescence of droplets in microfluidic 3D-printed devices; and physicochemical data for selected naphthenic acids (PDF)
Video showing the initial and final chambers of the microfluidic device with O/W emulsions inside (MP4)
