# Supplementary material for: A Hydrophilic 3D-Printed Microfluidic Device for Emulsion Studies: Preliminary Observations on the Role of Naphthenic Acids in Coalescence
Source: ACS Omega. 2025 Nov 5;10(45):53998–4008. doi: 10.1021/acsomega.5c05121 (PMC12631662; doi:10.1021/acsomega.5c05121)
Supplement: Supplementary file 1 [file ao5c05121_si_001.pdf]

Supporting Information

A Hydrophilic 3D-Printed Microfluidic Device for Emulsion Studies: Preliminary Observations on the Role of Naphthenic Acids in Coalescence

Lucas Paines Bressan[1], Reverson Fernandes Quero[1], Millena Couto dos Santos[1], Rogério Mesquita de Carvalho[2] and Leandro Wang Hantao[1,3,4,5*]

[1]Instituto de Química, Universidade Estadual de Campinas.

[2]Centro de Pesquisas Leopoldo Américo Miguez de Mello (CENPES), Petrobras – Rio de Janeiro, RJ, Brasil.

[3]Instituto Nacional de Ciência e Tecnologia (INCTBio) – Campinas, SP, Brasil.

[4]Centro de Estudos de Energia e Petróleo (CEPETRO).

[5]Núcleo Interdisciplinar de Planejamento Energético (NIPE) – Campinas, SP, Brasil.

* Corresponding author.

E-mail address: wang@unicamp.br (L. W. Hantao)

Table of Contents

Figure S1 – Mechanical tests performed on the developed hydrophilic resin. 7
Figure S2 – Characterization tests performed on the developed 3D-printing resin. 7
Figure S3 – Schematic representation of different regions of the proposed 3D-printed microfluidic device...8
Figure S4 – Histograms of the relative frequency of particles as a function of equivalent diameter. 8
Figure S5 - The time series images show different stages of the coalescence process of oil droplets 9

Table S1 – Composition of saline waters used in the coalescence study. 2
Table S2 – Weight increase (%) after 24 hours of immersion. 4
Table S3 – Comparison of commercially available resins and the one used in this work 3
Table S4 – Review of some works in the literature relating the generation of droplets using 3D-printed materials 5
Table S5 – Physico-chemical data for selected naphthenic acids. Data was obtained from Chemicalize (ChemAxon, Budapest, Hungary) 10

Table S1 – Composition of saline waters used in the coalescence study.

Constituent	Saline Water 1 (mg/L)	Saline Water 2 (mg/L)
Sodium (Na)	27,214	27,090
Potassium (K)	310	553
Magnesium (Mg)	356	95
Calcium (Ca)	722	112
Barium (Ba)	84	60
Strontium (Sr)	209	70
Chloride (Cl)	43,732	40,703
Bromide (Br)	0	211
Sulfate (SO_4)	122	41
Bicarbonate (HCO_3)	239	100
Acetate ($C_2H_3O_2$)	0	0
pH	7.4	8.3

Table S2 – Comparison of commercially available resins and the one used in this work.

Resin & Manufacturer	Price (per kg/L)	Achievable Resolution (Manufacturer Claim)	Resistance to Organic Solvents	Transparency / Optical Properties	Website
Liqcreate Bio-Med Clear (Liqcreate)	~\$55 - \$90	Layer height: 25-100 µm; High accuracy.	Resistant to ethanol.	Clear, translucent.	liqcreate.com
Formlabs Clear Resin V4.1 (Formlabs)	~\$159	Print resolutions: 25, 50, and 100 µm layers.	24h weight gain: Acetone (3.1%). Data for toluene/xylene not provided.	Polishes to near optical transparency. VLT: 85% at 2 mm thickness.	formlabs.com
B9Creations HD Clear (B9Creations)	~\$209	High detail; channel diameters as fine as 100-350 µm.	Data not provided by manufacturers. Safety Data Sheet lists incompatibilities with peroxides, strong bases, and oxidizing agents.	Translucent; can be made fully transparent with polishing or coating.	shop.b9c.com
CADworks3D Clear Microfluidics Resin V7.0a (CADworks3D)	Quote-based	Can achieve 50 µm for open channels.	Data not provided by manufacturers. Safety Data Sheet indicates an acrylate-based composition.	Clear, low viscosity resin.	cadworks3d.com
EnvisionTEC E-Shell 300 (EnvisionTEC)	Quote-based	High-resolution, material for hearing aid applications.	Water-resistant. Detailed organic solvent resistance is not provided.	Clear, transparent. Available in multiple transparent tones.	specialchem.com
Resin used in this work (Polaris Microsystems & Nanotechnology)	Research only	Can achieve closed channels of 40 µm	Resistant to organic solvents such as xylene and toluene	Transparent, hydrophilic without further modification, low viscosity, easy to clean	polarisnano.com.br

Table S3 – Weight increase (%) after 24 hours of immersion.

**Solvent**	**Hydrophilic Resin**
Butyl acetate	0.11%
Acetone	2.29%
Acetic Acid (5%)	0.12%
Hydrochloric Acid (pH = 0)	0.12%
Water	<0.10%
Saltwater (3.5% NaCl)	<0.10%
Isopropyl Alcohol	0.13%
Diesel	0.11%
Tripropylene Glycol Monomethyl Ether	0.15%
Hexane	0.18%
Sodium Hydroxide (pH = 10)	0.11%
Isooctane	0.11%
Hydraulic Oil	0.12%
Mineral Oil	0.13%
Hydrogen Peroxide (3%)	0.14%
Xylene	0.14%

Table S4 – Review of some works in the literature relating to the generation of droplets using 3D-printed materials

Title	3D-Printer	Resin	Was the microfluidic device 3D-printed?	Droplet generation of oil in water?	Coalescence study?	Ref.
Rapid assembly of multilayer microfluidic structures via 3D-printed transfer molding and bonding	ProJet™ 3000	VisiJet EX200 (structural epoxy)	No, the molds were 3D-printed. The final device was made of PDMS cast from these molds (3D-Printed Transfer Molding).	No.	No.	1
3D printed fittings and fluidic modules for customizable droplet generators	Form 2™ (Formlabs)	Formlabs Clear Resin	No, only the fittings and holders were 3D-printed. The device was assembled from these parts, commercial needles, and tubes.	Yes, the paper states the axisymmetric device is capable of generating both oil-in-water (O/W) and water-in-oil (W/O) droplets.	No.	2
3D free-assembly modular microfluidics inspired by movable type printing	nanoArch® S130 and P140	BIO (biocompatible resin)	No, modular molds were 3D-printed. The final device was made of PDMS by casting from the assembled molds.	No, the paper demonstrated the generation of water-in-oil droplets.	Yes, a 'fishbone' structure was designed and used to facilitate the merging of alternating droplets.	3
A 3D-printed modular magnetic digital microfluidic architecture for on-demand bioanalysis	Stereolithography (SLA) and Fused Deposition Modeling (FDM) printers were used.	Clear resin for SLA and Acrylonitrile Butadiene Styrene (ABS) filament for FDM.	Yes, the baseboard and functional modular components that form the digital microfluidic platform were directly 3D-printed and assembled.	No, the platform manipulates pre-existing aqueous droplets; it does not generate emulsions.	Yes, the paper demonstrated the merging of two droplets containing different quantum dots and analyzed the subsequent mixing efficiency.	4
3D-printed microfluidic device for monodisperse emulsions preparation	Ultimaker 2+ (Fused Filament Fabrication)	Not resin. CPE (Copolyester) filament	Yes, the frame and the interchangeable microfluidic chips containing the channels were directly 3D-printed.	Yes, the generation of both water-in-oil (W/O) and oil-in-water (O/W) droplets.	No. Surfactants were used specifically to prevent droplet coalescence.	5

Fabrication of 3D printed modular microfluidic system for generating and manipulating complex emulsion droplets	Projet MJP 2500 plus (Inkjet 3D Printing)	VisiJet M2R-CL (UV curable polymer resin)	Yes, individual functional modules (e.g., droplet generators) were directly 3D-printed and then assembled into a complete system.	Yes, generation of oil-in-water (O/W) emulsions.	Yes, an electrode module was used to induce electrocoalescence.	6
A Monolithic 3D Printed Axisymmetric Co-Flow Single and Compound Emulsion Generator	Form 3 (Stereolithography)	Clear Resin (light-reactive thermoset resin)	Yes, the entire device was fabricated as a single, monolithic piece using a 3D printer.	Yes, generation of oil-in-water (O/W) emulsions.	No. Surfactants were used to stabilize emulsions and prevent coalescence.	7
Our work	LCD-based SLA printer	Custom-made hydrophilic resin	Yes, fully printed with sealed microfluidic channels	Yes, without need to change material surface	Yes, coalescence study applied to petroleum emulsions of oil in water and naphthenic acid influence without external actuators	This study

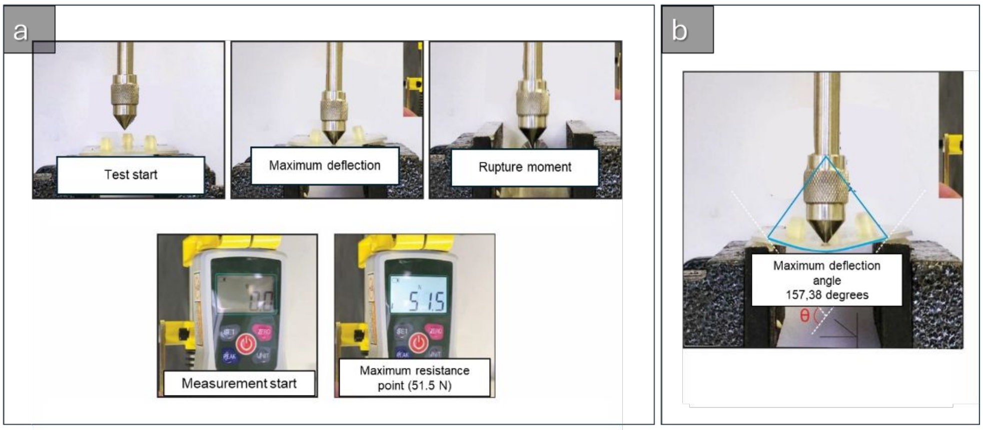

Figure S1 – Mechanical tests performed on the developed hydrophilic resin. a) Resistance measurement and its results; b) Maximum deflection angle measurement.

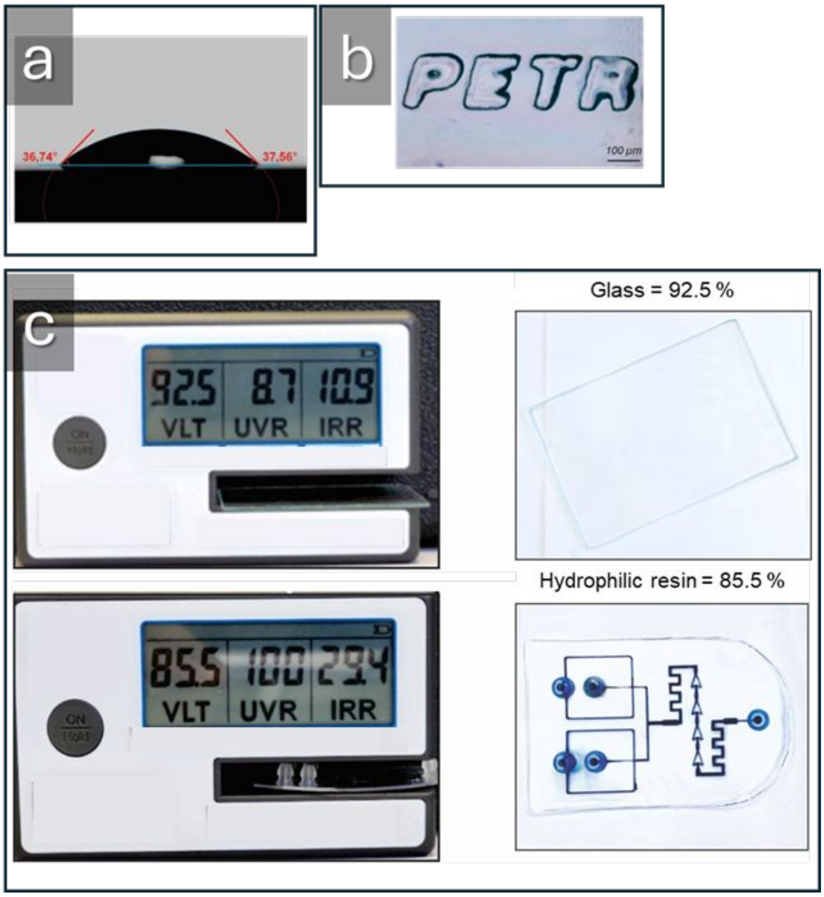

Figure S2 – Characterization tests performed on the developed 3D-printing resin. a) Contact angle result for the hydrophilic resin. b) 3D-Printed "PETR" letters using the developed resin showcasing the resolution obtained. c) Visible light transmittance (VLT) measurements of both glass and a device printed using the hydrophilic resin.

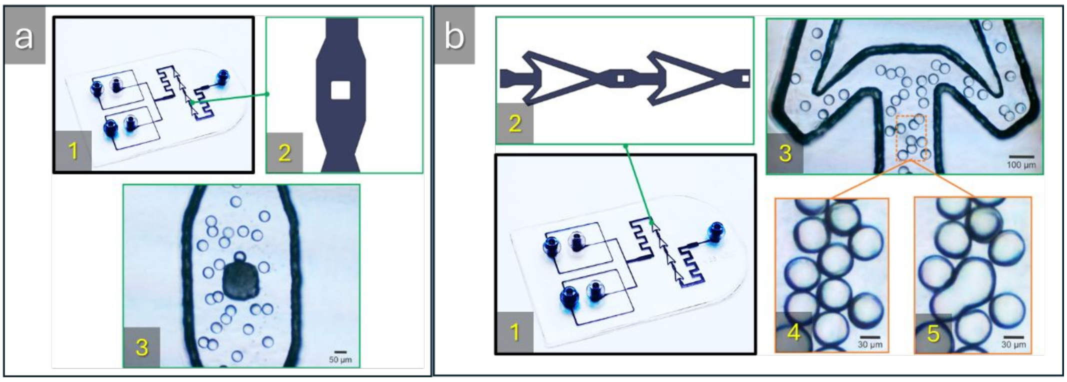

Figure S3 – Schematic representation of different regions of the proposed 3D-printed microfluidic device. a) Representation of the region specifically designed to organize droplets. 1) 3D-printed device with the region for droplet organization highlighted. 2) Schematic representation of the microfluid channel. 3) Microscopic image of the droplets in the designed region. b) Representation of the region specifically designed to promote coalescence. 1)The 3D-printed device with the region highlighted. 2) Schematic drawing of the microfluidic channels. 3) Microscopic image of the region for the coalescence of droplets, where 4) shows the moment before coalescence and 5) droplets coalescing.

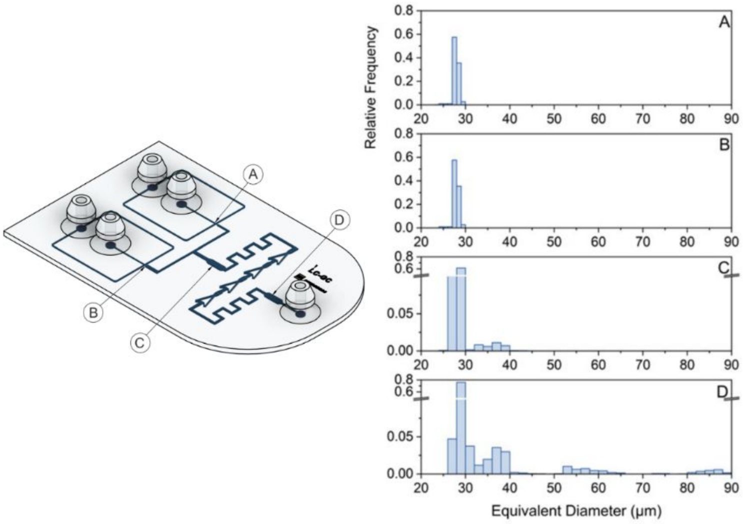

Figure S4 – Histograms of the relative frequency of particles as a function of equivalent diameter, measured in micrometers (µm), for four distinct regions of the device (A, B, C, D).

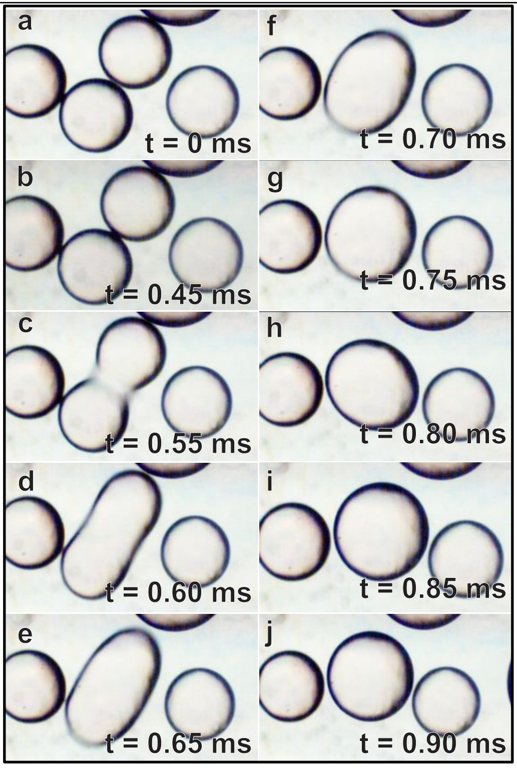

Figure S5 - The time series images show different stages of the coalescence process of oil droplets. (a) Initial state showing separate oil droplets before the coalescence process begins. (b) Droplets start to converge with visible deformation at the point of nearest approach. (c) Increased deformation of droplets due to closer proximity and interfacial forces. (d) Droplets continue to deform and move closer, beginning the process of thin film drainage. (e) The thin film between droplets drains further, causing the droplets to flatten at the interface. (f) (g) (h) (i) Droplets approach final coalescence with the liquid bridge dominating the inter-droplet space. (j) Completion of the coalescence process resulting in a single, larger oil droplet.

Table S5 – Physico-chemical data for selected naphthenic acids. Data was obtained from Chemicalize (ChemAxon, Budapest, Hungary)

Name	Carbon Number	DBE	Solubility at pH 7.4 (mg/mL)	Solubility at pH 8.3 (mg/mL)	pKa	HLB
Cyclopentene-carboxylic acid	6	3	550.70	550.66	4.73	7.818
Cyclohexane-carboxylic acid	7	2	231.80	231.78	4.82	6.561
Cyclohexaneacetic acid	8	2	142.20	142.20	4.94	5.999
5-Cyclohexylpentanoic acid	11	2	166.20	166.22	5.10	4.566
4-Cyclohexylbut-2-ynoic acid	11	3	5.102	40.352	3.50	5.063
Adamantane-carboxylic acid	11	4	89.41	180.25	4.62	4.609
Undecanoic acid	11	1	4.452	35.251	4.95	4.545
Myristic acid	14	1	0.1502	1.1894	4.95	3.333

References

(1) Glick, C. C.; Srimongkol, M. T.; Schwartz, A. J.; Zhuang, W. S.; Lin, J. C.; Warren, R. H.; Tekell, D. R.; Satamalee, P. A.; Lin, L. Rapid Assembly of Multilayer Microfluidic Structures via 3D-Printed Transfer Molding and Bonding. *Microsyst Nanoeng* **2016**, *2* (1), 16063. https://doi.org/10.1038/micronano.2016.63.

(2) Vijayan, S.; Hashimoto, M. 3D Printed Fittings and Fluidic Modules for Customizable Droplet Generators. *RSC Adv* **2019**, *9* (5), 2822–2828. https://doi.org/10.1039/C8RA08686A.

(3) Huang, S.; Wu, J.; Zheng, L.; Long, Y.; Chen, J.; Li, J.; Dai, B.; Lin, F.; Zhuang, S.; Zhang, D. 3D Free-Assembly Modular Microfluidics Inspired by Movable Type Printing. *Microsyst Nanoeng* **2023**, *9* (1), 111. https://doi.org/10.1038/s41378-023-00585-1.

(4) Kanitthamniyom, P.; Zhou, A.; Feng, S.; Liu, A.; Vasoo, S.; Zhang, Y. A 3D-Printed Modular Magnetic Digital Microfluidic Architecture for on-Demand Bioanalysis. *Microsyst Nanoeng* **2020**, *6* (1), 48. https://doi.org/10.1038/s41378-020-0152-4.

(5) Klusák, J.; Mucha, J.; Večeř, M. 3D-Printed Microfluidic Device for Monodisperse Emulsions Preparation. *Chemical Papers* **2021**, *75* (11), 6101–6113. https://doi.org/10.1007/s11696-021-01782-w.

(6) Song, R.; Abbasi, M. S.; Lee, J. Fabrication of 3D Printed Modular Microfluidic System for Generating and Manipulating Complex Emulsion Droplets. *Microfluid Nanofluidics* **2019**, *23* (7). https://doi.org/10.1007/s10404-019-2258-2.

(7) Ghaznavi, A.; Lin, Y.; Douvidzon, M.; Szmelter, A.; Rodrigues, A.; Blackman, M.; Eddington, D.; Carmon, T.; Deych, L.; Yang, L.; Xu, J. A Monolithic 3D Printed Axisymmetric Co-Flow Single and Compound Emulsion Generator. *Micromachines (Basel)* **2022**, *13* (2), 188. https://doi.org/10.3390/mi13020188.
